# Inhibition of Glycogen Synthase Kinase 3β Promotes Tight Junction Stability in Brain Endothelial Cells by Half-Life Extension of Occludin and Claudin-5

**DOI:** 10.1371/journal.pone.0055972

**Published:** 2013-02-13

**Authors:** Servio H. Ramirez, Shongshan Fan, Holly Dykstra, Slava Rom, Aaron Mercer, Nancy L. Reichenbach, Larisa Gofman, Yuri Persidsky

**Affiliations:** 1 Department of Pathology and Laboratory Medicine, Temple University School of Medicine, Philadelphia, Pennsylvania, United States of America; 2 Center for Substance Abuse Research, Temple University School of Medicine, Philadelphia, Pennsylvania, United States of America; 3 Department of Molecular and Integrative Physiology, University of Michigan, Ann Arbor, Michigan United States of America; University Heart Centre Freiburg, Germany

## Abstract

Neuroinflammatory conditions often involve dysfunction of the Blood-Brain Barrier (BBB). Therefore, identifying molecular targets that can maintain barrier fidelity is of clinical importance. We have previously reported on the anti-inflammatory effects that glycogen synthase kinase 3β (GSK3β) inhibition has on primary human brain endothelial cells. Here we show that GSK3β inhibitors also promote barrier tightness by affecting tight junction (TJ) protein stability. Transendothelial electrical resistance (TEER) was used to evaluate barrier integrity with both pharmacological inhibitors and mutants of GSK3β. Inhibition of GSK3β produced a gradual and sustained increase in TEER (as much as 22% over baseline). Analysis of subcellular membrane fractions revealed an increase in the amount of essential tight junction proteins, occludin and claudin-5, but not claudin-3. This phenomenon was attributed to a decrease in TJ protein turnover and not transcriptional regulation. Using a novel cell-based assay, inactivation of GSK3β significantly increased the half-life of occludin and claudin-5 by 32% and 43%, respectively. A correlation was also established between the enhanced association of β-catenin with ZO-1 as a function of GSK3β inhibition. Collectively, our findings suggest the possibility of using GSK3β inhibitors as a means to extend the half-life of key tight junction proteins to promote re-sealing of the BBB during neuroinflammation.

## Introduction

The blood brain barrier (BBB) shields the brain parenchyma from immune cells and toxins in the blood, thus maintaining the adequate environment needed for normal neuronal and glial cell function [1]. Compared to other capillary endothelium, brain endothelium has specialized characteristics, such as tight junctions, specialized transport systems, and lack of fenestrate [2]. Under normal physiological conditions, the role of the BBB is to protect and maintain the delicate neuronal environment. Neuroinflammation resulting from a cerebrovascular accident, neurological disorder, infectious disease or brain trauma, causes disruption of the BBB and leaves the CNS vulnerable to neuronal damage [3]–[4]. Therefore strategies that aid in restoration of BBB integrity could greatly improve neurological outcomes [5]. One manifestation of BBB dysfunction is evident by increased permeability of blood solutes into the brain parenchyma which is greatly controlled by the tight junction (TJ) complex located between endothelial cells. This physical barrier is mainly responsible for generating the hallmark features of the BBB. The TJs restrict the paracellular movement of solutes (water soluble and polar compounds) and small ions providing the brain endothelium with high transendothelial electrical resistance (TEER) [2]. At the molecular level, the following proteins are highly enriched at the TJ complex: occludin, claudin (claudin-3, 5, 12), zonula occludens proteins (ZO-1, -2) and the junctional adhesion molecules (JAMs) [2]. The assembly of the TJ is such that the intracellular ZO-1, -2 proteins form the major protein docking site for transmembranous occludin, claudin and JAMs. The TJ complex is not a rigid structure. The dynamic nature of the TJ in response to cellular stimuli can manifest as disassembly, re-distribution, degradation and remodeling [6]. These events impact barrier genesis, barrier maintenance and barrier dysfunction (seen in neuropathological conditions).

Glycogen synthase kinase 3β (GSK3β) is a serine/threonine kinase initially identified as the final enzyme involved in the glycogen synthesis metabolic pathway. However, the role of GSK3β has expanded to also include regulation of cell division, differentiation, apoptosis, signal transduction, and inflammation [7]. In resting cells, GSK3β is active, but can be inactivated upon phosphorylation of its Ser9 residue by various kinases (p90Rsk, p70S6 kinase, AKT, certain isoforms of PKC and PKA) [8]. Inactivation of GSK3β can also occur by forming protein complexes with specific molecular binding partners (as in the case of WNT signaling) or by blocking the ATP-binding site with a pharmacological inhibitor [9]. The anti-inflammatory effects resulting in GSK3β inhibition have been shown *in vitro* and in several *in vivo* models of acute and chronic inflammation [10], [11], [12]. Specific to brain endothelial cells, the anti-inflammatory role of GSK3β has also been previously described [12]. Our previous work indicated that GSK3β inhibition in primary human brain microvascular cells (BMVEC) reduced adhesion/migration of primary human monocytes across BMVEC monolayers, diminished expression of pro-inflammatory factors in brain endothelium and attenuated BBB disruption (TEER decrease) during monocyte-BMVEC interaction [12]. These observations prompted us to look into the effects of GSK3β inhibition on BBB function under physiologic conditions. Indirect evidence gives support to the notion that GSK3β is involved in BBB function. Observations using pulmonary endothelial cells has indicated that GSK3β inhibition resulting from hepatocyte growth factor signaling promote endothelial barrier properties [13]. In addition, investigations in the developing mouse embryo have revealed a putative role for GSK3β in barriergenesis (via canonical WNT signaling) [14]. Conversely, GSK3β activation has been implicated in causing a “leakier” BBB in mice deficient of platelet endothelial cell adhesion molecule (PECAM-1), developing a more severe experimental autoimmune encephalomyelitis [15].

Although GSK3β has numerous substrates, β-catenin is of particular interest in the context of the BBB. Normally, GSK3β phosphorylates β-catenin, making it a target for ubiquitin-proteosomal degradation; inactivation of GSK3β, therefore, allows β-catenin to accumulate [16]. β-catenin has both a structural role (i.e., via interactions with cadherin) and a co-transactivator of transcription role. As a co-transactivator, β-catenin associates with the lymphoid enhancer factor (LEF)/T-cell factor (TCF) family of transcription factors and mediates expression of target genes involved in differentiation, polarization and cell fate [17]. Recently, claudin-1, -2 and -3 (which are found at the TJ) have been identified as target genes for β-catenin/LEF/TCF [14], [18], [19]. However claudin-5, occludin, and ZO-1 gene regulation do not appear to be induced by the β-catenin transcriptional complex [14]. An indirect mechanism for occludin regulation by β-catenin has been described. In this case, it was shown that β-catenin shuttling to adherens junctions, instead of LEF/TCF binding, downregulated the transrepressor, Slug (which inhibits transcription of occludin in epithelial cells) [20], [21]. Although the role of β-catenin as a structural molecule and co-transactivator has been well studied in epithelial junctions, its role in brain endothelial TJ remains largely unexplored, in particular, whether structural β-catenin contributes to the stabilization of brain endothelial tight junctions.

Here we show that inhibition of GSK3β in primary human BMVEC enhances barrier tightness as evaluated by TEER. This effect was demonstrated using GSK3β-specific pharmacological inhibitors and by overexpression of mutants of GSK3β. Also, using a novel assay for determining claudin-5 and occludin turnover, we determined for the first time the half-lives of these two proteins in brain endothelial cells. Importantly, this assay platform revealed that GSK3β inhibition significantly extends the half-life of both claudin-5 and occludin. Also, our analysis implicates the structural, and not the transactivation role, of β-catenin in contributing to the stabilization of TJ proteins. Together, these results highlight the possible use of GSK3β inhibitors in BBB repair as well as the utility of a novel cell-based assay for examining changes in tight junction protein stability.

## Materials and Methods

### Reagents and Cell Culture

The following GSK3β inhibitors were used: lithium chloride (LiCl) and kenpaullone (Ken) were purchased from Sigma-Aldrich (St. Louis, MO) and 3-(2,4-dichlorophenyl)-4-(1-methly-1H-indol-3-yl)-1H-pyrrole-2,5-dione (SB216763) was purchased from Enzo Life Sciences (Plymouth Meeting, PA).

Primary cultures of human BMVEC (provided by Michael Bernas and Dr. Marlys Witte, University of Arizona, Tucson, AZ) were isolated from microvessels derived from temporal or hippocampal tissue removed during operative treatment of epilepsy as described [22]. Tissue used for BMVEC isolation was outside epileptogenic foci. The procedures were approved by the Temple University Institutional Review Board. BMVEC cultures were expanded and maintained in DMEM/F-12 media containing 10% fetal bovine serum, endothelial cell growth supplement (BD, Franklin Lakes, NJ), heparin (1 mg/ml, Sigma, St. Louis, MO), amphotericin B (2.5 µg/ml), penicillin (100 U/ml), and streptomycin (10 µg/ml). BMVEC cultures were used at low passage (between 2 and 5). Unless otherwise stated, reagents used for cell culture were purchased from Life Technologies (Carlsbad, CA). The human embryonic kidney cell line HEK293 was obtained from ATCC (Manassas, VA) and cultured in Dulbecco’s modified Eagle’s minimal essential medium (DMEM) with 10% fetal bovine serum, 2 mM Glutamax and 50 units/ml penicillin/50 µg/ml streptomycin. For use with the TJ protein stability system which requires doxycycline, the standard serum was replaced with 10% tet-system approved FBS from Clontech Laboratories (Mountain View, CA).

### Real Time qPCR

The gene expression profile of TJ proteins from various donors was performed by qPCR. Total RNA was isolated using the RNAqueous-4PCR DNA-free RNA Isolation kit (Life Technologies). RNA purity and concentration were determined with a NanoDrop ND-1000 spectrophotometer (Thermo Fisher Scientific, Fair Lawn, NJ). Conversion to cDNA was performed by reverse transcription using 2 µg of total RNA with the High-Capacity cDNA Reverse Transcription Kit (Applied Biosystems, Foster City, CA). The cDNA (diluted 1∶20) template was then mixed with both the Maxima Probe/ROX qPCR Master Mix (Fermentas, Hanover, MD) and the corresponding human TaqMan Gene Expression Assay (CLD1: Hs01076359_m1, CLD3: Hs00265816_s1, CLD5: Hs01561351_m1, OCC: Hs00170162_m1, ZO-1: Hs01551876_m1, ZO-2: Hs00910541_m1 and JAM-2: Hs00221894_m1 from ABI) according to the manufacturer’s instructions; for internal controls, human TaqMan gene expression pre-developed assays for GAPDH and RPLPO (Applied Biosystems) were also used. QPCR was performed on an Applied Biosystems StepOnePlus Real-Time PCR System. Raw data were analyzed with DataAssist software (Applied Biosystems) using the ΔΔCt method (relative quantification). Results are expressed in relative gene expression levels (fold) compared to the untreated control.

### Measurement of Barrier Function by Transendothelial Electrical Resistance (TEER)

Measurement of TEER on BMVEC monolayers used to determine the effect of GSK3β inhibition on barrier integrity was performed using the 1600R electric cell-substrate impedance system (ECIS) (Applied Biophysics, Troy, NY) as previously described [22]. The ECIS system provides real-time monitoring of changes in TEER. In brief, BMVEC at 1×10^5^/well were plated on collagen type I coated 8W10E+ electrode arrays (Applied Biophysics). The cells were allowed to form monolayers until stable TEER values were reached. After seven days (with media change every three days), the monolayers were exposed to the indicated GSK3β inhibitor or recombinant adenoviruses expressing GSK3β mutants. Readings were acquired continuously at 4000 Hz at 30 min intervals. Confluent BMVEC monolayers demonstrated baseline TEER readings between 1000–2000 Ω. The data is presented as the percent change from baseline TEER ± SEM from at least three independent experiments containing three condition replicates.

### Plasmid Construction and Recombinant Adenoviral Vectors

To generate the fusion protein constructs, human claudin-5 or occludin were fused with green fluorescence protein (AcGFP) at its N-terminus (shown as AcGFP- CLD5 and AcGFP-OCC) Claudin-5 and occludin encoding open reading frame (ORF) were amplified from claudin-5 (hereafter CLD5) (CLDN5 transcript; NM_003277) Human cDNA ORF Clone (ORIGENE. #RC207122) and occludin (hereafter OCC) (OCLN transcript; NM_002538) Human cDNA ORF Clone (ORIGENE. #RC206468) with primer pairs (ggactcagatctatggggtccgcagcgttggag and aaagcggtaccctaga cgtagttcttcttgtcgtag for CLD5 and cactcttcttcgagcaatgtcatccaggcctcttg and tggttgatatcatg tcatccaggcctcttgaa for OCC, respectively), then the PCR products were digested and subcloned into cloning vector pAcGFP1-C1 (Clontech Laboratories Inc, Mountain View, CA) to form pAcGFP-CLD5 and pAcGFP-OCC. The insert sequences in all pAcGFP constructs were verified by DNA sequencing. These fusion constructs were then cloned with a modified second generation Tet-Off system from Clontech. pTRE-AcGFP-IRES-mCherry, pTRE-AcGFP-CLD5-IRES-mCherry, and pTRE-AcGFP-OCC-IRES-mCherry were generated by first cloning mCherry (from pmCherry, Clontech), into the multiple cloning site (MCS) B of pIRES (Clontech). Then AcGFP-X, where X represents not fused or fused to CLD5 or OCC, was cloned into the MCS A of the pIRES-mCherry construct. Next, the pCMV IE promoter of the AcGFP-X-IRES-mCherry was replaced with the TRE-tight promoter from pTRE-Tight-Bi (Clontech). All vector cloning used standard digestion/ligation subcloning techniques. To use pTRE-AcGFP-X-IRES-mCherry, the transactivating vector containing the tTA-advanced (Clontech) must also be transfected into the cells. Addition of doxycycline turns off gene expression by preventing the transactivator from binding to the TRE promoter.

Adenoviruses expressing either the GSK3β S9A or GSK3β KM mutants were a kind gift from Dr. Stephen Dewhurst (University of Rochester Medical Center, Rochester, NY). The construction of these adenoviral vectors was done as previously described [8]. Recombinant adenoviruses were generated following transfection of HEK293A cells and purified using the Adeno-X Maxi purification system from Clontech.

### Western Blotting and Immunoprecipitations

BMVEC cell monolayers were treated and lysed to produce either whole cell or subcellular fractions. For preparation of whole cell lysates, the CelLytic-M cell lysis reagent (Sigma) supplemented with a 1∶500 dilution of a broad specificity protease inhibitor cocktail (Sigma) was used. Subcellular fractions (cytosolic, membranous, or nuclear) were collected using the Subcellular Protein Fractionation Kit (Thermo Scientific) or ProteoExtract Subcellular Proteome Extraction Kit (Calbiochem, San Diego, CA). The BCA protein assay (Thermo Scientific) was then used to determine the protein content in the fraction. For immunoprecipitation, fractions containing 500 µg in total protein were precleared with protein A-Sepharose beads (Santa Cruz Biotech, Santa Cruz, CA) for 1 hr and incubated with antibodies to ZO-1 at 4°C overnight. The immunoprecipitated complexes were washed 5 times with RIPA buffer. Proteins from either the immunoprecipitated products or from the membrane fractions (15–30 µg of total protein) were mixed with 6X loading buffer containing DTT and then boiled for 10 min at 95°C. The proteins were then separated by SDS-PAGE (4–20% precast gels) (Thermo Scientific), followed by electrophoretic transfer to nitrocellulose membranes. The primary antibodies were diluted in 1X TBS/0.1% Tween 20 and used to detect the following proteins: anti-occludin (diluted 1∶500) from US Biologicals (Swampscott, MA); anti-claudin-5 (diluted 1∶300); anti-claudin-1 (diluted 1∶300); anti-claudin-3 (diluted 1∶300) from Life Technologies; anti-β-catenin (diluted 1∶300) from Santa Cruz Biotech; anti-GSK-3β (diluted 1∶500); anti-lamin A/C (diluted 1∶500) from Cell Signaling Technology (Danvers, MA); anti-HA.11 (diluted 1∶500) from Covance (Princeton, NJ). All primary antibodies were incubated with the membranes overnight at 4°C with gentle shaking. Species-specific peroxidase-conjugated secondary antibodies (diluted 1∶1000) (Thermo Scientific) were incubated with the membranes for 1 hr at room temperature. Proteins were detected using the Supersignal West-Femto chemiluminescent substrate (Thermo Scientific) with a G:Box Chemi HR16 (Syngene, Frederick, MD) gel documentation system.

### Transient Transfection and Imaging

HEK293 cells plated at a density of 1×10^6^ were transfected with the inducible constructs for occludin or claudin-5 using Lipofectamine LTX with Plus reagent (Life Technologies) according to the manufacturer’s instructions. After 24 hr, cells were maintained either without doxycycline or exposed to doxycycline (200 ng/ml) to turn off transgene expression. Transfection into BMVEC was performed by electroporation using two pulses of 1400 v and 20 s with the Neon® transfection system (Life Technologies) according to the manufacturer’s instructions. Of note, the use of the Neon® transfection system on BMVEC allows for 60–80% transfection efficiency (depending on passage and donor). After 24 hr, cells were placed in media with or without doxycycline.

Imaging of transfected cells expressing AcGFP (either fused to the TJ protein or alone) and mCherry from the bicistronic construct (see plasmid construction section) were visualized using a live cell imaging system (Carl Zeiss MicroImaging, Thornwood, NY), consisting of an Axio Observer Z1 microscope configured for widefield epi-fluorescence fitted with an AxioCam HR camera. Image acquisition and analysis was performed using AxioVision (v4.7) imaging software from Carl Zeiss MicroImaging.

### siRNA-mediated GSK3β Knockdown

BMVEC were transfected at 2×10^6^ cells per ml with a pool of siRNAs to GSK3β (ON-Target plus® smart pool, catalog number L-003010-00-0005, Thermo Scientific, Dharmacon, Lafayette, CO) or with a pool of control siRNAs, (Accell non-targeting siRNA pool, catalog number D-001910-01, Thermo Scientific, Dharmacon) at different concentrations (0, 75, 100 and 150 nM) with the Neon® Transfection System (Life Technologies) using the same settings as described above under transient transfection. Cells were collected 48, 72 and 96 hr after transfection and processed for either whole cell lysate or cytosolic and membrane fraction preparation.

### TCF/LEF Luciferase Reporter Assay

BMVEC were grown to 90% confluence in 96-well plates and then transduced with a lentivirus containing the Cignal Lenti TCF/LEF reporter, Cignal Lenti CMV-Renilla (internal control) or the Cignal Lenti CMV-GFP (transfection efficiency control) using the SureENTRY Tranduction Reagent (SA Biosciences, Frederick, MD) according to the manufacturer’s protocol. After 48 hr, the cells were treated with GSK3β inhibitors (as indicated in the figure) for 24 hr. The cells were harvested and assayed for luciferase activity using the Dual-Luciferase Reporter Assay System (Promega, Madison, WI). Readings were obtained using an Infinite 200 PRO luminometer (Tecan Group Ltd., Männedorf, Switzerland).

### Statistical Analysis

Results are presented as mean ± SD or SEM and p values <0.05 are considered significant. Data were analyzed using the Prism v5 software (GraphPad, La Jolla, CA) and statistical significance determined by performing unpaired two-tailed Student’s t test or ANOVA with the Dunnett’s post test.

## Results

### Inhibition of GSK3β Increases Barrier Tightness

**Figure 1:**
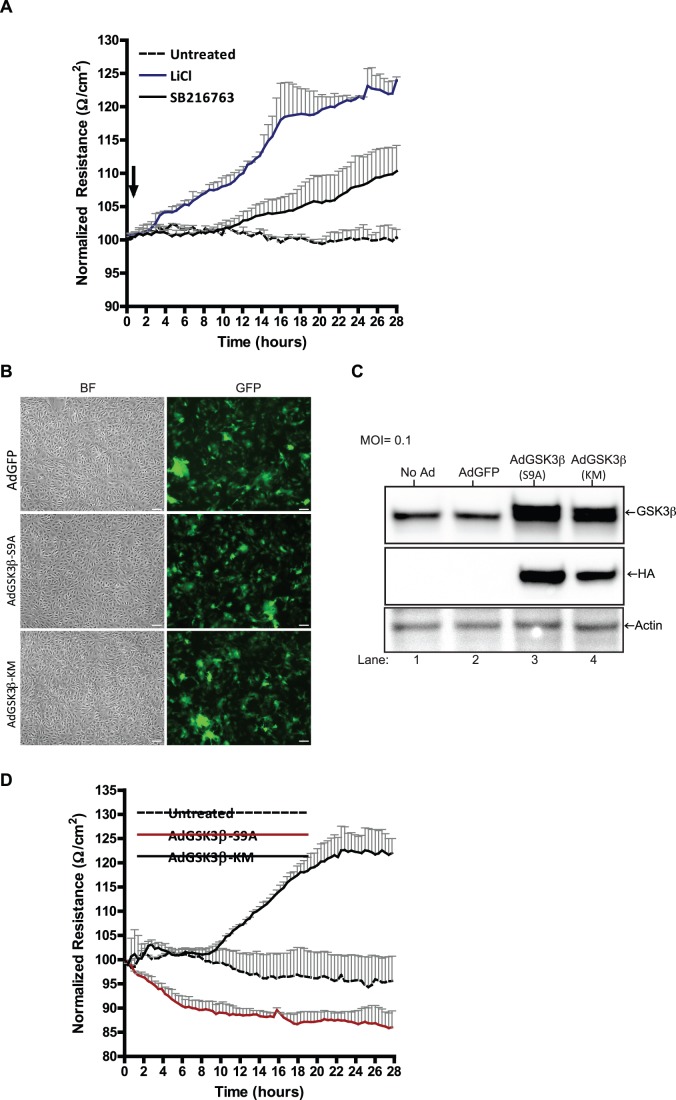
GSK3β inhibition affects barrier function. (A) Trans-Endothelial Electrical Resistance (TEER) was measured continuously in primary human brain microvascular endothelial cells (BMVEC) with or without GSK3β inhibitors LiCl (10 mM) and SB216763 (20 µM). After baseline TEER was achieved (between 7–10 days), the inhibitors were added at the time point indicated by the arrow. The resistance was measured at 4000 Hz in 20-min intervals for the duration of the time shown. (B) Representative images from BMVEC exposed to adenoviral vectors expressing the GSK3β mutants. The vectors are bicistronic for GFP which allows for monitoring of transduction efficiency which was greater than 80% (5X objective power magnification; scale bar:100 microns). (C) After 24 hr post-infection, transduced BMVEC were lysed and western blots examined for over-expression of mutant protein. Adenovirus-mediated gene transfer resulted in high expression of GSK3β mutants (lanes 3 and 4). The detection of human influenza hemagglutinin (HA) peptide tag fused to the GSK3β mutant denotes the specific expression level of the transgene. (D) TEER measurements (∼18 hr post infection) were performed on BMVEC transduced at a multiplicity of infection (MOI) of 0.1 with adenovirus-expressing mutants of GSK3β. AdGSK3β-KM indicates the dominant negative form of GSK3β containing a mutation in the kinase domain. AdGSK3β-S9A expresses the active form of GSK3β which prevents inactivation by phosphorylation on the serine 9 residue. TEER values are represented as the average (line) normalized TEER with the positive SEM (n = 3).

Previous studies have indirectly demonstrated the effects of GSK3β inhibition on barrier function [13], [14]. However, no determination was made whether the barrier enhancing effect observed was specific to GSK3β inhibition. Therefore, using TEER to measure barrier function, we examined whether the addition of two different classes of GSK3β inhibitors would affect barrier tightness. BMVEC monolayers (plated on ECIS microelectrode arrays) were treated with GSK3β inhibitors, LiCl (10 mM) and SB216763 (20 µM), and TEER was monitored over the course of 28 hr (Figure 1A). Values for untreated represents the steady state baseline TEER after BMVEC monolayers have become confluent and TJ were formed. Inactivation of GSK3β by LiCl addition gradually increased the average TEER by 10% at 12 hr and 22% at 24 hrs above baseline resistance (Fig. 1A). SB216763, an ATP competitive inhibitor of GSK3β, also increased TEER, but in a delayed fashion when compared to LiCl, reaching a maximum TEER increase of 7% at 24 hr. To confirm that the effect seen on TEER was due to inhibition of GSK3β and not other cellular targets, we next evaluated the effect of GSK3β mutants on TEER. BMVEC were transduced at a multiplicity of infection (MOI) of 0.1 with replication-deficient adenoviruses encoding the previously characterized GSK3β S9A and GSK3β KM mutants [8], [23]. Apart from encoding the GSK3β mutant, these bicistronic vectors also express GFP, thus providing visualization of an infection efficiency of ∼80% (Fig. 1B). AdGSK3β-S9A expresses a constitutively active mutant, whereas the AGSK3-KM expresses a catalytically inactive mutant. At 24 hr post-infection, transduced BMVEC were lysed and examined for over-expression of the mutant protein as indicated by higher levels of GSK3β (Fig. 1C top panel) and the hemagglutinin (HA) tag which is only present on the transgene (Fig. 1C middle panel). Next, TEER measurements (shown at 18 hr post infection) were performed on BMVEC expressing the mutants of GSK3β protein (Fig. 1D). Consistent with observations with GSK3β inhibitors, the kinase inactive (KM) form resulted in steady TEER enhancement, providing a 24% increase over the basal reading. However, the active form (S9A) of GSK3β produced a 13% decline in TEER. Of note, no deleterious effects on barrier function were observed by the addition of replication deficient adenovirus at the indicated MOI. Adenovirus encoding GFP alone had similar baseline TEER readings as the uninfected control (data not shown). Therefore, the effects observed by inactivation of GSK3β with pharmacological inhibitors are specific because biochemical inactivation also produced comparable results. Together, these analyses suggest that inactivation of GSK3β in brain endothelial cells directly benefits barrier integrity.

### GSK3β Increases the Levels of TJ Proteins in the Membrane Fraction

**Figure 2:**
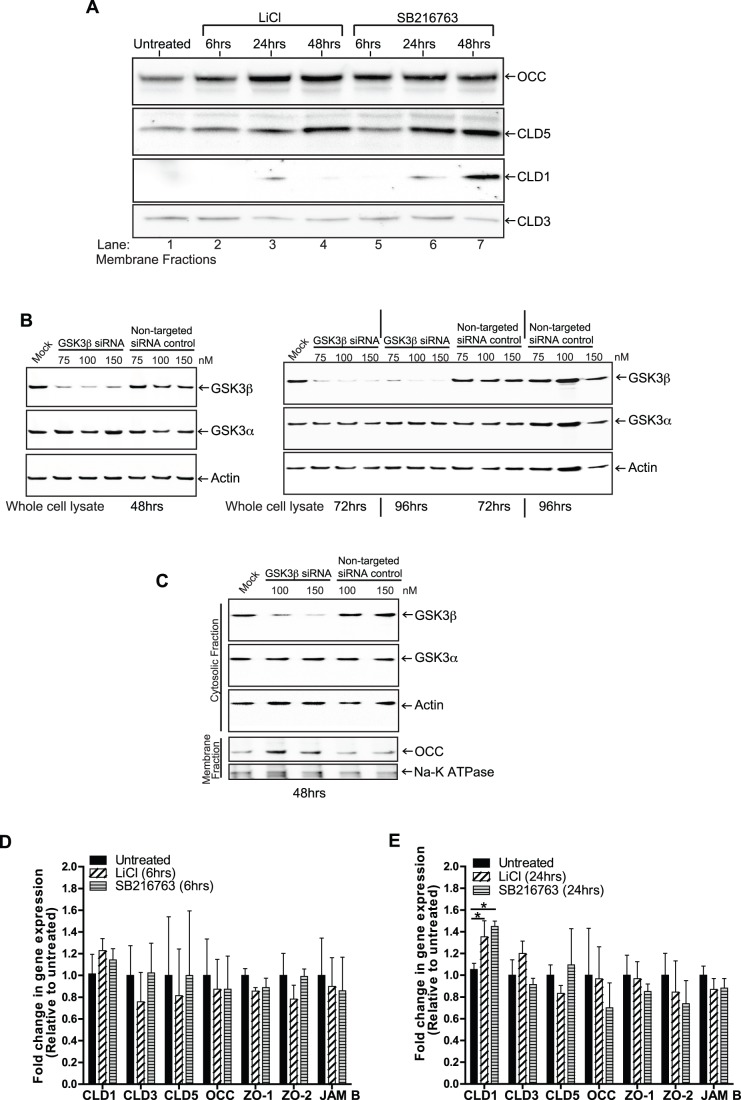
GSK3β inhibition regulates the presence of tight junction proteins in BMVEC membrane fractions. (A) Western blots of BMVEC membrane fractions from cells treated for 6, 24 and 48 hr with GSK3β inhibitors LiCl (10 mM) and SB216763 (20 µM). Except for claudin-3 (CLD3), the levels of occludin (OCC), claudin-1 (CLD1) and claudin-5 (CLD5) increased in response to the GSK3β inhibitors. (B, C) Western blot analysis of BMVEC cell lysates (B) and cellular fractions (C) after transfection with no siRNA (mock), non-targeted and GSK3β targeting siRNAs. Efficient knock-down of GSK3β, and not GSK3α, resulted in increased levels of occludin (C). Note: actin and the Na-K ATPase were used as loading controls. (D and E) Total RNA was extracted from BMVEC (treated as described in panel A) and the mRNA levels of the indicated genes were determined by quantitative PCR at (D) 6 hr and (E) 24 hr. The housekeeping genes, GAPDH and RPLP0, were used as endogenous internal reference genes. The data was analyzed using the ΔΔCt method and the results are expressed as relative gene expression levels (fold regulation) comparing the treated with the untreated control. The results are shown as the average+SD fold regulation from three different BMVEC donors.

Next, we investigated whether the effect on TEER upon inhibition of GSK3β could be a result of increased levels of TJ proteins. Membrane fractions from BMVEC treated for 24 hr with GSK3β inhibitors, LiCl (10 mM) or SB216763 (20 µM), were analyzed for the amount of TJ proteins, occludin (OCC), claudin-1 (CLD1), claudin-3 (CLD3) and claudin-5 (CLD5). As shown in Fig. 2A, inactivation of GSK3β with either inhibitor produced a time-dependent increase in both occludin and claudin 5. Levels of occludin were increased as early as 6hrs, maximizing at 24 hr and remaining unchanged at 48 hr. When compared to untreated cells, claudin-5 showed a marginal increase at 6 hr, continuing at 24 hr and maximizing at 48 hr. Interestingly, levels of claudin-1 were undetected in resting BMVEC, but were detected at 24 hr after LiCl treatment and after both 24 and 48 hr treatment with SB2216763. The levels of membranous claudin 3 were unaffected by either treatment any time point. These results suggest that GSK3β influences barrier tightness by affecting two of the primary proteins present in brain endothelial TJ.

To ensure that the effects on TJ protein expression are the result of GSK3β inhibition and not due to inhibition of the GSK3α isoform, BMVEC were trasfected with a pool of 4 siRNAs targeting GSK3β. Transfection of increasing amounts of siRNAs showed efficient knockdown of GSK3β at 48, 72 and 96 hr. Analysis by densitometry pointed to at least 85% knockdown with the highest amount of targeted siRNAs at all time points (Fig. 2B) tested. As expected, GSK3β, and not GSK3α, was markedly decreased by the targeted siRNAs. In addition, neither the mock transfected or the non-targeted pool of siRNAs affected the levels of GSK3β or GSK3α. Since significant levels of GSK3β could effectively be reduced 48 hr post GSK3β siRNA transfection, subsequent transfections at this time point were used for evaluation of TJ proteins. Figure 2C shows representative western blot results of cellular fractions after siRNA mediated knock-down of GSK3β. As can be observed, occludin levels (in membrane fractions) were greatly upregulated in the experimental conditions with targeted siRNA. Similar results as those observed for occludin were also obtained for claudin-5 (data not shown). Taken together, these results strongly suggest that the effect on TJ proteins observed with pharmacological inhibition of GSK3 is likely a consequence of GSK3β rather than GSK3α.

One possibility that could explain the increase in TJ proteins after inhibition of GSK3β may involve the transcriptional induction of genes encoding for TJ proteins. To test this notion, BMVEC from multiple donors were treated with LiCl or SB216763 for 6 hr and 24 hr and profiled for gene expression of: OCC, CLD-1, -3, -5, ZO-1, ZO-2 and JAMB (Fig. 2D and 2E). We used the ΔΔCt method and fold-regulation to compare treated versus untreated BMVEC. The results showed no statistical significance in gene regulation for most of the examined genes coding for TJ proteins. The only exception was claudin-1, which showed a slight increase when GSK3β inhibitors were present. Similar to the levels registered with the membrane protein extracts, GSK3β inhibition did appear to affect claudin 1 gene regulation which showed a statistically significant increase at 24 hr but not at 6 hr. This was a surprising finding given that the effect on TEER is likely due to the prominent increase on TJ protein levels (membrane fractions, Fig. 1A) of occludin and claudin-5. Re-distribution of protein from the cytosolic to the membranous compartment did not appear to be a contributing factor in GSK3β inhibition (data not shown) because cytosolic TJ protein content did not significantly change. Collectively, these results suggest that the effect of GSK3β inhibition on TEER involving TJ protein increase in membrane fractions is not due to the gene regulation of occludin or claudin-5.

### A Novel Cell-based System to Evaluate Tight Junction Protein Stability

**Figure 3:**
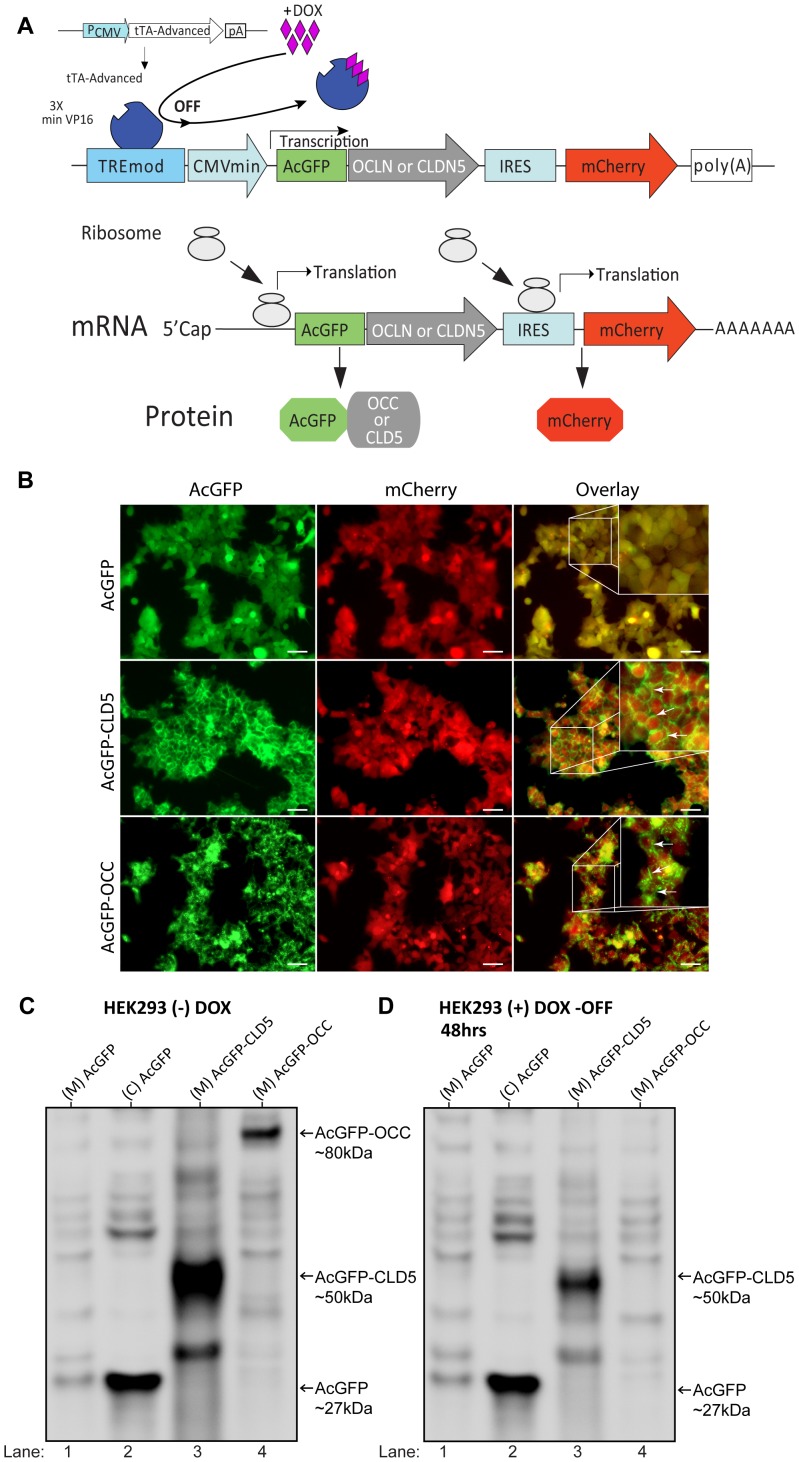
Cell-based assay for evaluating tight junction protein stability. (A) Schematic diagram of the “Tet-off” based TJ protein stability system. The system is designed to use reporter constructs which express bicistronic mRNA under the control of a tetracycline inducible promoter. The first cassette expresses the fusion protein of AcGFP with human claudin-5 or occludin at the N-terminus. The second cassette expresses mCherry red fluorescence protein after the internal ribosome entry site (IRES). In the induced state, the Tet-transactivator protein (tTA) activates transcription by binding to the TRE element. In the un-induced state, the addition of doxycycline (Dox) prevents the tTA protein from binding to the TRE promoter region, thereby blocking transcription. Once turned off, the level of protein turnover can be determined from the ratio of the GFP fused protein to mCherry as a function of time. (B) Representative images from HEK293 cells transfected with the tight junction protein stability system constructs. The top left panel shows the cytosolic expression of unfused AcGFP. The middle and lower left panels show the membranous expression of AcGFP-CLD5 and AcGFP-OCC, respectively. The cytosolic expression of mCherry is shown for all constructs (middle column). Original objective magnification was 20X and 60X (insert), scale bars:50 microns. (C) Western blots probed for GFP showing the expression of the fused tight junction proteins without (left) and with doxycycline (Dox, 200 ng/ml) for 48 hr. The expression of unfused AcGFP is observed in the cytosolic fraction (lane 2) and not in the membrane fraction (lane 1). The levels of membrane-bound AcGFP-CLD5 and AcGFP-OCC shown in the membrane fraction are depleted when in the presence of doxycycline. (M) indicates membrane fraction; (C) indicates cytosolic fraction.

Given that GSK3β inhibition did not induce redistribution or transactivation of occludin or claudin-5 genes, experiments were conducted to evaluate whether protein half-life was affected. To explore this possibility, we developed an in vitro cell-based assay for analyzing occludin and claudin-5 protein turn over using methodology previously described for the global protein stability system [24]. The system offers various advantages compared to traditional methods for measuring protein turnover including: real-time monitoring by either FACS or microscopy, assessment at the single cell level and high-throughput capability [24]. The system employs reporter constructs which express bicistronic mRNA under the control of an inducible promoter (a TetOff system). The reporter construct contains two expression cassettes from one mRNA transcript. The first cassette expresses human claudin-5 or occludin fused with green fluorescence protein (AcGFP) at its N-terminus, shown as CLD5-AcGFP and OCC-AcGFP, respectively. The second cassette expresses the mCherry protein, derived from *Dicosoma* protein DsRed, after the Internal Ribosome Entry Site (IRES) (Fig. 3A). In the TetOFF system, the Tet-Advanced transactivator (tTA) protein binds to the tetracycline response element (TRE) promoter region to allow transactivation of the transgenes. Addition of the tetracycline analog, doxycycline (Dox), binds to tTA and prevents binding to the TRE. When in the induced state, both mCherry and AcGFP-CLD5 or -OCC are produced at a constant ratio because the expression originates from the same mRNA. Addition of Dox halts transactivation and allows protein turnover to be monitored as function of time. To validate the system, HEK293 cells were transfected with the inducible constructs expressing either AcGFP, AcGFP-CLD5 or AcGFP-OCC (Fig. 3B) and visualized after 24 hr. As expected, transfection with the vector coding for unfused AcGFP causes accumulation of AcGFP in the cytosolic compartment (Fig. 3B, top panel). The reference reporter, mCherry, also appears in the cytosol. However, transfection with vectors that express the AcGFP-CLD5 or AcGFP-OCC localizes the tethered tight-junction protein to the membrane (Fig. 3B, middle and bottom panel, respectively). To confirm transgene regulation, transfected cells with or without the addition of Dox were lysed after 48 hr and then evaluated by immunoblotting against AcGFP (Fig. 3C). Fractions from cells without Dox show protein expression from the three transgenes having the corresponding molecular weights for the AcGFP unfused and fused TJ proteins. As visualized by microscopy, only the AcGFP fused to the TJ protein appears in the membrane fractions; (M) indicates membranous and (C) indicates cytosolic (Fig. 3C). Exposing the cells to 200 ng/ml of Dox turns off gene expression and thus a significant decrease in the fused TJ protein is observed (Fig. 3D). As can be seen in the immunoblot, no significant reduction in the unfused AcGFP was noted when Dox was present (Fig. 3D) which demonstrates that AcGFP has slow turnover kinetics affected only when fused to the TJ protein. Therefore, this inducible cell-based assay for two key tight junction proteins found on brain endothelium could be used to evaluate whether GSK3β inhibitors affect tight junction protein stability.

### GSK3β Inhibitors Increase Occludin and Claudin-5 Protein Stability

**Figure 4:**
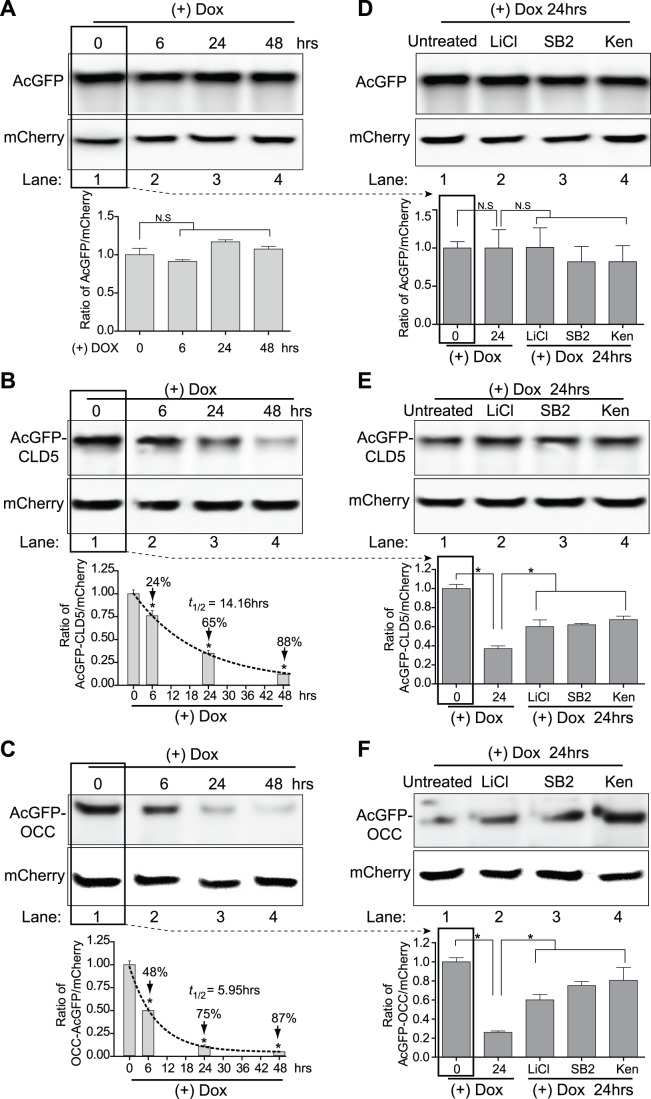
Evaluation of TJ protein stability with GSK3β inhibitors in HEK293 cells. Panels A-F show western blots of HEK293 cells expressing the tight junction protein stability system after treatment with doxycycline to turn off transgene expression. The cells were monitored at 0, 6, 24 and 48 hr, lysed and western blots were probed for GFP and mCherry. (A) The expression of unfused AcGFP is shown by densitometry analysis. (B and C) The fused AcGFP-CLD5 and AcGFP-OCC protein degradation is shown as a function of time. The values in the graph represent the densitometric values from the ratio of fused protein to mCherry normalized against the zero time point. To determine the protein turnover half-life, the values were fitted to first order kinetics (dashed line). The results are shown as the mean+SEM (n = 3). (*) denote a difference of P<0.05 between the groups compared (bracket) or to the zero time point. The effect of GSK3β inhibitors LiCl (10 mM), SB216763 (SB2, 20 µM), kenpaullone (Ken, 2 µM) on AcGFP, AcGFP-CLD5 and AcGFP-OCC expression are shown in panels D-F, respectively. After addition of Dox for 24 hr, the cells were lysed and western blots for GFP were performed. Basal turnover is shown in lane 1; the inhibition in tight junction protein turnover resulting from GSK3β inhibition is shown observed in lanes 2–4. The graphed densitometric values were calculated using the ratio of AcGFP to mCherry with normalization to fold change. Note: the densitometry shows the time zero reference which is not shown in the blots (see B and C). The results are shown as the mean ± SEM (n = 3), * denotes a difference of P<0.05 between the groups compared (bracket).

Using the developed assay for TJ protein stability system, the effects of three GSK3β inhibitors were examined. First, a time course from 0 to 48 hr was used to evaluate the normal turnover rate of occludin and claudin 5 in transfected HEK293 cells. Consistent with Figure 3D, unfused AcGFP was stable throughout the time course and showed no significant degradation (Fig. 4A). In the case of AcGFP-CLD5, protein levels decreased by 24%, 65% and 88% at 6, 24 and 48 hr, respectively (Fig. 4B). Application of first order kinetics from the densitometry measurements after normalization to mCherry showed a calculated half-life for AcGFP-CLD5 of approximately 14.16 hr. Running the same experimental parameters for AcGFP-OCC revealed a decrease in protein of 48%, 75% and 87% at 6, 24 and 48 hr, respectively (Fig. 4C). Estimation of the turnover of AcGFP-OCC provided a half-life of approximately 5.95 hr. Next, the turnover of occludin or claudin-5 was determined in response to LiCl and two cell permeable GSK3β inhibitors (Fig. 4E and 4F). The following GSK3β inhibitors were used for 24 hr: LiCl (10 mM), SB21676 (20 µM) and kenpaullone (2 µM). Analysis with the claudin-5 TJ protein stability system showed a significant attenuation in protein turnover with the addition of LiCl which resulted in 40% loss of protein compared to 63% in the untreated (Fig. 4E). The GSK3β ATP binding competitors SB21676 and kenpaullone also showed similar results of about 40% (Fig. 4E). In the case of occludin, the stability system indicated that GSK3β inhibition had a more pronounced effect on the protein turnover (Fig. 4F). With these optimized concentrations of GSK3β inhibitors, we demonstrated a 75% protein loss for untreated compared to only 25% with either of the inhibitors tested (Fig. 4F), therefore the protective effect on occludin was 3 fold when compared to untreated. Thus collectively, the results from the TJ protein stability system strongly indicate that GSK3β inhibition affects protein turnover kinetics thereby offering an explanation as to how modulation of GSK3β impacts tightness of the BBB.

### GSK3β Inhibitors Increase Tight Junction Protein Stability in Primary Human Brain Microvascular Endothelial Cells (BMVEC)

**Figure 5:**
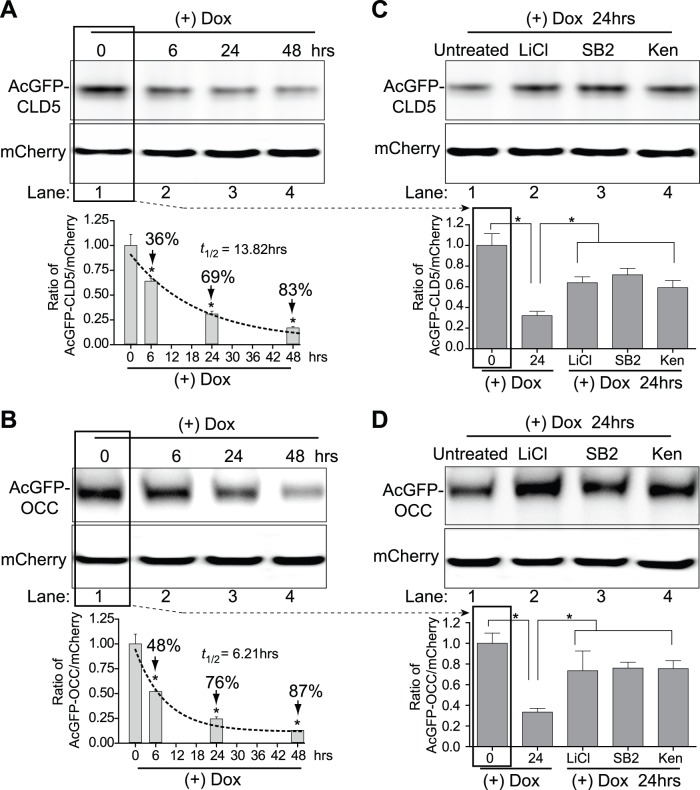
GSK3β inhibition regulates occludin and claudin-5 stability in BMVEC. BMVEC were transfected with the tight junction stability system for 24 hr and 200 ng/ml of doxycycline (Dox) was added for 6, 24 or 48 hr. Panels A and B: Basal protein turnover of AcGFP fused claudin-5 (A) and occludin (B) was evaluated by western blots probed for GFP. Graphed results from densitometric analysis are shown along with protein half-life determinations using first order kinetics (dashed line). The values are expressed as the mean ± SEM (n = 3) of the normalized densitometry ratio of the fused protein to that of co-expressed reference protein mCherry. (C and D) Show the effect of GSK3β inhibitors on claudin-5 (C) and occludin (D) protein turn-over at 24 hrs using the tight junction protein stability system. The untreated controls display the marked reduction in tight junction protein at 24 hrs while those with GSK3β inhibitors significantly attenuate this effect. The graphed densitometry values were calculated using the ratio of AcGFP to mCherry and normalized to fold change. Note: the densitometry shows the time zero reference which is not shown in the blots (see A and B). The results are shown as the mean ± SEM (n = 3), (*) denotes a difference of P<0.05 between the groups compared (bracket).

Although GSK3β inhibition using the TJ protein stability system in HEK293 cells showed remarkable attenuation in claudin-5 and occludin protein loss, it is possible, however, that the results may not translate to the brain endothelial cell. Therefore, the constructs for the TJ protein stability system were transfected into BMVEC and analysis for half-life was performed. As shown in Figure 5A, the basal turnover of the GFP-fused claudin-5 in transfected BMVEC had a half-life of approximately 13.82 hr, a comparable 21 min difference between transfected HEK293 cells and BMVEC. The fusion construct for occludin indicated a basal half-life of approximately 6.21 hr, a rate of degradation similar to the 5.95 hr observed for HEK293 cells, a difference of 16 min; Fig. 5B). Confirming the results with HEK293 cells, BMVEC also showed less degradation of TJ proteins in the presence of GSK3β inhibitors. Transfection with the stability system resulted in claudin-5 TJ protein that was degraded approximately 68% at 24 hr, but only 36% when either of the inhibitors was introduced (Fig. 5C). Interestingly, greater attenuation in protein turnover was found with AcGFP-OCC, providing approximately 67% loss of occludin, whereas GSK3β inhibition allowed only 24% loss (Fig. 5D). Performing the analysis of degradation in BMVEC using the TJ stability system pointed to similar protein kinetics as in the HEK293 cells. More importantly, both types of GSK3β inhibitors (ATP and non-ATP competitors) indicated that TJ protein stability can be achieved by regulating GSK3β activation. Of note, the acquired occludin and claudin-5 half-lives using the TJ protein stability system were comparable (within an hour) to those determined by the traditional pulse-chase method (data not shown).

### TJ Protein Stability is Affected by β-catenin at the Cell Membrane and not by Gene Regulation

**Figure 6:**
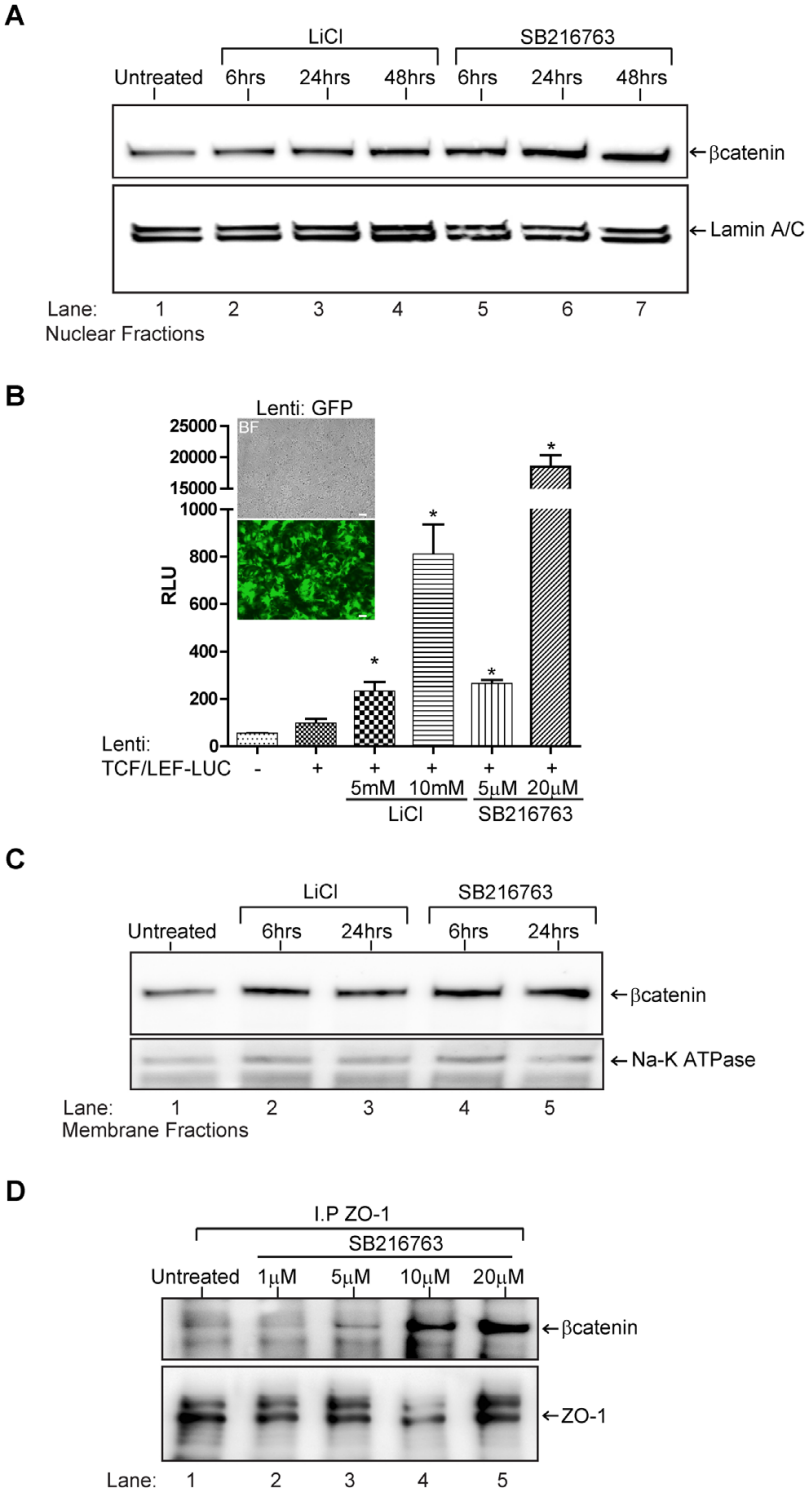
Effects of GSK3β inhibition on transcriptional and structural β-catenin in BMVEC. (A) Western blots of BMVEC nuclear fractions show a time-dependent increase in β-catenin accumulation in cells treated with LiCl (10 mM) or SB216763 (20 µM); lamin A/C denotes the loading control. (B) Results from luciferase assays of BMVEC transduced with lentiviruses containing the TCF/LEF sensitive luciferase reporter. A significant increase of the luciferase reporter is evident in BMVEC treated with the indicated GSK3β inhibitor (concentrations are the same as above) for 24 hr. Representative images showing lentiviral transduction efficiency (∼90%) in BMVEC using a lentiviral construct expressing a GFP reporter (insert, 5X objective magnification, scale bars: 100 microns). The luciferase results are expressed in relative light units (RLUs) after normalization with measurements provided by a co-infected (lentivirus) renilla luciferase internal control. The values are shown as the mean ± SEM (n = 3), * denotes a difference of P<0.05 when compared to the transduced but untreated condition. (C) Western blots from BMVEC membrane preparations showing membrane bound β-catenin after cells were exposed to LiCl or SB216763 (concentrations as above) for 6 and 24 hr (Na/K ATPase serves as the loading control). (D) Enhanced β-catenin association with ZO-1 observed by co-immunoprecipitation analysis. BMVEC monolayers were incubated without or with escalating doses (1–10 µM) of SB216763. Proteins were extracted under native conditions, immunoprecipitated (I.P.) for ZO-1 and immunoblotted (I.B.) for β-catenin.

GSK3β is known to have a number of protein substrates; however, no other substrate has been better characterized to be a primary substrate than β-catenin [25]. Under resting conditions, active GSK3β phosphorylates cytosolic β-catenin which then targets its ubiquitination and degradation. Upon inhibition of GSK3β, β-catenin accumulation can re-distribute to the nucleus or near the membrane. Therefore, we tested whether the observed barrier tightness and TJ protein stability could be a function of the status of β-catenin. First, analysis for the presence of β-catenin in the nucleus was performed to evaluate the nuclear redistribution of β-catenin after GSK3β inhibition. Absence of β-catenin in the nuclear compartment may be possible in light of the lack of transcriptional regulation of the occludin and claudin-5 genes. BMVEC were treated with either LiCl (10 mM) or SB216763 (20 µM) for the indicated times (Fig. 6A). Nuclear fractions were then collected and probed for β-catenin. As shown, a clear and progressive increase in β-catenin accumulation was observed (Fig. 6A). Therefore, it does not appear that inhibition of GSK3β in BMVEC affects the expected accumulation of β-catenin in the nucleus. Experiments were also performed to evaluate the transcriptional role of β-catenin. To this end, BMVEC were transduced with a lentiviral TCF/LEF-luciferase reporter. These results indicate that β-catenin, although transcriptionally active upon GSK3β inhibition, does not affect occludin or claudin-5 gene expression (Fig. 2D and 2E).

As stated earlier, β-catenin also serves a structural role in the cell; it interacts with cytoskeletal proteins and members of adherent and TJ complexes. Therefore, it could be reasoned that β-catenin accumulation may redistribute to the TJ in brain endothelial cells, thereby promoting stability of the TJ proteins. To address this possibility, membrane fractions from BMVEC were analyzed for increased β-catenin expression following GSK3β inhibition by LiCl and SB216763. As shown in Figure 6C, β-catenin was significantly increased both at 6 and 24 hr. Aside from being membrane-bound, β-catenin association (immunoprecipitation) with ZO-1 was also increased in relationship to escalading doses of GSK3β inhibitor (Fig. 6D). As a consequence of GSK3β inhibition, β-catenin immunoprecipitation with the TJ protein scaffold ZO-1 suggests that its added incorporation stabilizes the TJ complex at the BBB.

## Discussion

To date, the role of GSK3β on brain endothelial barrier function remains largely unknown. Here, using primary human brain microvascular endothelial cells (BMVEC), we have performed a detailed study analyzing the effects of GSK3β inhibition on TJ protein expression. We demonstrate that inactivation of GSK3β leads to a gradual and sustained increase in tightness of the barrier. This effect was observed with both cell permeable pharmacological inhibitors and over-expressed mutants of GSK3β. Significantly, the inhibition of GSK3β in BMVEC consistently increased the levels of the essential TJ proteins, claudin-5 and occludin, on membrane fractions. Moreover, the effect of GSK3β on membrane enrichment of claudin-5 and occludin was not the result of de novo synthesis, but rather of protein stabilization. Using a novel assay (presented here for the first time) for evaluating TJ protein expression, we report that inhibition of GSK3β considerably extends the half-life of occludin and claudin-5. These findings offer insight into possible therapeutic avenues for stabilizing the TJ and improving barrier function at the BBB.

As the gatekeeper of the CNS, the BBB conceptualizes the presence of an immunological, transporter, metabolic and physical barrier [1]. These barrier mechanisms allow for proper microenvironment composition that is essential for synaptic communication. We have previously reported on the anti-inflammatory effects that GSK3β inhibition has on brain endothelial cells [12]. Specifically, the inactivation of GSK3β in stimulated brain endothelium decreased the secretion of pro-inflammatory cytokines and the expression of adhesion molecules, ICAM-1 and VCAM-1. Consequently, it was found that the “immunological barrier” was maintained because GSK3β inhibition significantly reduced immune-endothelial interaction and leukocyte transendothelial migration. In this report, we tested whether GSK3β also has a role in impacting the “physical barrier” (i.e., the TJ complex) of the brain endothelium. We began by studying the transendothelial electrical resistance (TEER) to evaluate barrier integrity. TEER works on the principle of measuring the impedance generated from cells cultured on an electrode array for which a small alternating frequency (4000 Hz) has been applied [26], [27]. To a larger extent, the current flow moves under and in between the cells and thus provides an analytical measurement of the tightness of the barrier. Complex impedance allows both resistance and capacitance measurements to be derived. The analysis showed a gradual and sustained increase in TEER in monolayers of BMVEC upon exposure to GSK3β inhibitors SB216763 and LiCl. Interestingly, LiCl resulted in a quicker response, possibly due to a more rapid cellular diffusion compared to SB216763. These results complement a previous study performed in pulmonary artery endothelial cells where inhibition of GSK3β via hepatocyte growth factor (HGF) elevated TEER [13]. However, unlike pharmacological inhibition of GSK3β, the increase in TEER was short lived (lasting only a few hours) when HGF was placed on pulmonary endothelial cells, an effect the authors attributed to receptor desensitization. The effect of GSK3β at the BBB has also been explored indirectly, by activation of the wnt signaling cascade [14]. In the canonical wnt pathway, GSK3β inhibition can occur by the binding of wnt ligands to partnering frizzled receptors [16]. Once the wnt ligand and frizzled receptor complex is nucleated, LRP 5/6 is recruited and intracellular signaling is triggered. The consequence of the canonical wnt signaling results in inactivation of GSK3β and disassembly of GSK3β with axin and APC, thereby preventing the degradation of β-catenin. Liebner and colleagues elegantly showed the importance of wnt signaling for barriergenesis pre- and postnatally in β-catenin LacZ transgenic mice [14]. The study found that β-catenin accumulation (induced by wnt) in brain endothelial cells is high during BBB maturation, but is drastically reduced once the BBB is fully matured. Among the TJ proteins tested in isolated mouse brain endothelial cells, the study also demonstrated that Wnt3a ligand induction of β-catenin leads to the increase of gene and protein expression of only claudin-3. Although our study uses human brain endothelial cells and pharmacological inhibitors of GSK3β, similar to Liebner et al., we found that there was no effect on the gene expression of TJ proteins, except for one of the claudins. In our case, only claudin-1 gene expression was increased at 24 and 48 hr post GSK3β inactivation. However, this distinction could be due to the manner in which GSK3β is inhibited or simply a difference from the species used.

Studies by Zhang and colleagues have provided some insight into the effect GSK3β inhibition has on TJ complex assembly [28]. Their analysis performed on the kidney epithelial cell line, MDCK, indicated that AMPK activation via the nectin-afadin system allows for greater ZO-1 membrane deposition. The study also showed that GSK3β inhibition induces accumulation of ZO-1 at the tight junctions; a process not dependent on E-cadherin. Although GSK3β inhibition in MDCK cells was shown to induce deposition of tight junction components at the plasma membrane, the investigation presented did not extend to β-catenin nor the effect of GSK3β on specific TJ proteins. Interestingly in stark contrast, a recent report also in MDCK cells shows that the opposite effect on tight junction complexes is observed when GSK3β is inhibited in epithelial cells [29]. In Stevenson et al., the study shows that instead of tightening of the epithelial barrier, GSK3β inhibition induces greater permeability. In the report the authors attribute the increase permeability on the effect of GSK3β on occludin, claudin-1 and E-cadherin down-regulated gene expression. It is likely that biochemical events occurring in epithelial cells differ from those in barrier forming endothelial cells thus bringing caution to relating findings of on cell type to the other. In fact, the results reported here does not show that GSK3β affects ZO-1 protein dynamics or the gene regulation of occludin in brain endothelial cells.

Our analysis of gene expression did not account for the increase in TEER or for the increase in the levels of claudin-5 or occludin in membrane fractions (seen as early as 6 hr) after addition of GSK3β inhibitors. This observation shifted our focus to protein turnover kinetics. In order to analyze claudin-5 and occludin protein turnover, we designed reporter constructs based on the global protein stability profiling system previously described by Yen and colleagues [24]. The system allows for the determination of protein stability from the ratio of a claudin-5 or occludin fused reporter to that of a second reporter expressed from a single mRNA transcript. Using this system, we determined the half-lives, under resting conditions, for these two TJ proteins. The half-life of occludin was determined to be approximately 6.2 hrs. To date, this is the first determination of occludin half-life in human brain endothelial cells. Prior studies in epithelial cell lines, LLC-PK1, IEC-6, and MDCK, have indicated an occludin half-life of 1.3, 2, and 11.2 hr, respectively [30]. This broad range may reflect differences in cell proliferation and TJ composition between epithelial cells and barrier forming endothelial cells. Our results are in close agreement with the half-life of 6.4 hr for human occludin determined by Raikwar et al. in HEK293 cells by the pulse-chase method [31]. Indeed, our vector system resulted in an occludin half-life of 5.95 hr in HEK293 cells, indicating that the assay presented here provides a measure of protein stability similar to those obtained with traditional methods. Importantly, the system revealed that the addition of GSK3β inhibitors greatly protected against occludin degradation, extending the occludin half-life 2.7 times (to ∼16 hr) when compared to untreated cells. As is the case with occludin, the half life of claudin-5 has not been previously determined in human brain endothelium. Thus far, the reported half-life of claudin-5 in endothelial cells has been reported to be 1.16 hr for HUVEC and >3 hr for bovine retinal endothelial cells [32], [33]. Our analysis points to a half-life of approximately 13.82 hr for claudin-5, giving support to the notion of the presence of cell-specific mechanisms in the regulation of claudin-5. Similarly to occludin, claudin-5 turnover was reduced by a factor of 1.8 in the presence of GSK3β inhibitors, extending the half-life to approximately 24 hr. It is important to mention that the half-life values shown in this report must be considered in the context of cell culture conditions. For our estimations in BMVEC, the cells were confluent in complete monolayers and were cultured in the absence of growth factors such as VEGF which was only used in the expansion phase of the cell culture (see methods and [22]). In fact Murakami and colleagues reported on the effect of VEGF on increasing occludin turnover [33]. Nonetheless, our results from both BMVEC and HEK293 suggest that the effects of inhibiting GSK3β should prove universal in the reduction of occludin and claudin-5 protein turnover.

Previously, Failor and colleagues used mammary epithelial tumor cells to show that the degradation of GSK3β by glucocorticoid stimulation induces β-catenin membrane localization which then increases transepithelial resistance [34]. Our study supports the above observation, and provides that this barrier enhancing effect is a consequence of TJ protein stability resulting from the structural role of β-catenin rather than its transactivational one. This assertion comes from the observation that occludin or claudin-5 gene regulation was not observed even though β-catenin was active in the nuclear fractions of BMVEC treated with GSK3β inhibitors. However, under the same treatment paradigm, we did find increased levels of β-catenin co-immunoprecipitated with the anchoring TJ protein, ZO-1, an association previously described in MDCK and also determined to be important in the formation of the blood-epididymal barrier [35], [36]. We propose that once the TJ complex is formed, the induced β-catenin can interact with ZO-1 to promote stabilization of TJ complex members, occludin and claudin-5. Admittedly, given the pleiotropic nature of GSK3β, it is unknown whether GSK3β inhibition affects the ubiquitination/proteosome degradation of occludin and claudin-5 or their lysosomal targeting. Studies of protein degradation have found that occludin binds to the itch E3 ubiquitin ligase which in turns targets its degradation [33], [37]. Thus far, ubiquitin ligases have not been shown to be direct substrates of GSK3β. However, indirect regulatory mechanism may be in place, as in the case of the novel ligase, atrogrin [38]. Atrogrin is a muscle-specific ubiquitin ligase that is upregulated by the transcription factor, FoxO. FoxO is a putative GSK3β substrate which upon phosphorylation by GSK3β enables its translocation to the nucleus [39], [40]. Therefore, GSK3β inactivation is implicated to reduce the mRNA levels of atrogrin, which then leads to the accumulation of proteins targeted by atrogrin. Less is known about claudin-5 degradation involving either the ubiquitin/proteosome pathway or lysosomal degradation. Only recently has the poly-ubiquitination of claudin-5 been described [32]. Poly-ubiquitination, a phenomenon strongly associated with proteosome mediated degradation, has been also shown for other members of the claudin family. However, it is unknown whether the E3 ubiquitin ligase LNX1p80 binds to claudin-5 as has been characterized for claudins -1, -2 and -4 [41]. Because occludin and certain claudins can be shuttled into lysosomes, future studies are needed to evaluate whether GSK3β affects lysosomal degradation of tight junction proteins.

In summary, we have demonstrated in human brain endothelial cells that enhanced barrier tightness can be achieved by the inactivation of GSK3β. The results shown provide evidence that inhibition of GSK3β leads to a decrease in claudin-5 and occludin protein turnover which does not involve their gene regulation. Furthermore, these analyses were acquired with the use of a newly developed vector-based molecular tool for evaluating TJ protein stability. The combination of GSK3β inhibitors as anti-inflammatory and barrier-enhancing agents gives promise to their utility in repair and protection of the blood-brain barrier.
